# Regulatory Assessment of Casgevy for the Treatment of Transfusion-Dependent β-Thalassemia and Sickle Cell Disease with Recurrent Vaso-Occlusive Crises

**DOI:** 10.3390/cimb46080485

**Published:** 2024-07-30

**Authors:** Essam Kerwash, Marija Sajic, Khadija Rerhou Rantell, James W. McBlane, John D. Johnston, Alison Niewiarowska, Andrew S. Butler, Susan Cole

**Affiliations:** Medicines and Healthcare Products Regulatory Agency (MHRA), 10 South Colonnade, London E14 4PU, UK

**Keywords:** sickle cell disease, transfusion-dependent β-thalassemia 2, Casgevy, CRIPSR/Cas9 technology

## Abstract

Sickle cell disease (SCD) and transfusion-dependent β-thalassemia (TDT) are hereditary haemoglobinopathies characterized by a reduction in functional β-globin chains. Both conditions cause tiredness and increase susceptibility to infection, which can lead organ failure, significantly reducing life expectancy and typically requiring those affected to undergo regular erythrocyte transfusion. Recently, a novel therapeutic treatment for SCD and TDT was approved by the UK regulatory body (Medicines and Healthcare products Regulatory Agency; MHRA). Exagamglogene autotemcel (Casgevy) is the first licensed therapy globally to utilize CRIPSR/Cas9 technology and induces an increase in expression of γ-globin chains to compensate for the reduction in functional β-globin. Casgevy represents a first-in-class therapeutic, and numerous considerations were made by the MHRA throughout its assessment of the medicine. These include, but are not limited to, the risk of tumorigenicity and off-target editing, a limited cohort size, the validity of proposed dosing and the conduction of only single-arm studies. The MHRA’s analyses of the data to support the proposed indications are presented and discussed throughout this manuscript. Overall, the sponsors claims were considered well supported by their data, and Casgevy was licensed for the treatment of TDT or SCD in patients 12 years of age and older for whom hematopoietic stem cell (HSC) transplantation is appropriate, but a human leukocyte antigen-matched related HSC donor is not available.

## 1. Introduction

### 1.1. Haemoglobin

Hemoglobin (Hb) is an essential molecule for the transport of oxygen and other gasses throughout the body. Functional Hb forms as a tetramer, with the adult form of hemoglobin (HbA) consisting of two α-globin chains and two β-globin chains [1,2]. During development, however, γ-globin is more highly expressed than β-globin, and therefore co-assembles with α-globin to form tetrameric fetal hemoglobin (HbF) [3,4]. HbF is critical in the relatively hypoxic in utero environment as it exhibits higher affinity for oxygen than HbA, allowing efficient transport of gasses across the placenta [5]. The phenotypic switch from HbF to HbA beings to occur after ~30 weeks of gestation, and by the end of an infant’s first postpartum year, the expression of HbF is reduced to <1%, making HbA the predominant form of Hb [3,4]. The developmental downregulation of γ-globin is regulated by a transcriptional repressor encoded by the *BCL11A* gene, and knockdown of this leads to an increase in HbF production [3,6].

### 1.2. Haemoglobinopathies

Mutations to the genes which encode α- (*HBA*) and β-globin (*HBB*) in adults result in the production of Hb with perturbed function or in the reduced production of Hb, and this can lead to severe and life-threatening anemia.

#### 1.2.1. Sickle Cell Disease

Sickle cell disease (SCD) represents a group of haemoglobinopathies which can trigger the deformation of red blood cells (RBCs) from their regular malleable, bi-concave disc shape into a hard sickle shape [3,7]. The most common and severe form of SCD is HbSS, in which a patient inherits two copies of the mutated form of the *HBB* gene (one from each parent; denoted as βS/βS genotype) [8]. The mutated form of Hb which is then produced, termed HbS, contains a valine-to-glutamic acid mutation at residue six in the β-subunit [7,8]. This substitution means that hydrophobic bonds can form between Hb tetramers, promoting the formation of polymeric Hb chains and consequently inflexible, sickle-shaped RBCs [3,9]. The extent of polymer formation is dependent upon Hb deoxygenation, the intracellular concentration of HbF and the intracellular concentration of HbS (hence heterozygous βS/βA carriers exhibit reduced symptoms compared homozygous βS/βS patients) [10,11].

Unlike the healthy, non-sickled RBCs, sickled RBCs agglutinate into clusters which can occlude the vasculature and lead to episodes of severe pain (vaso-occlusive crises) and, in the long-term, contribute to chronic organ damage and a reduction of life expectancy [12]. Other symptoms of SCD are anemia and an increased vulnerability to infection, alongside rarer complications such as gallstones, strokes, swelling of the spleen and eyesight problems [12,13]. It is noteworthy that in newborns prior to the completion of the phenotypic switch to HbA, and in adults with hereditary persistence of fetal hemoglobin, SCD presents with a less severe phenotype [14,15].

SCD predominantly occurs in people of African and African-Caribbean descent, likely as a result of evolutionary pressure, as the sickle cell trait is associated with protection against malaria. An estimated 8% of Black people are carriers for the sickle cell gene [12]. Current treatment typically relies upon managing symptoms with oral medication of hydroxyurea which promotes production of HbF, and this may be supported by treatment with crizanlizumab and/or voxelotor to help alleviate crises [12,13]. Additionally, blood transfusions can be used to reduce the proportion of RBCs containing high levels of HbS. It is recommended that transfusions are repeated every 3–6 weeks, and so can be burdensome for both patients and care trusts [12,13].

#### 1.2.2. Beta-Thalassemia

Thalassemia is an inherited haemoglobinopathy in which the quantity of produced Hb is significantly reduced compared with unaffected individuals. Alpha and beta variants occur due to mutations in the *HBA* and *HBB* genes, respectively, with these being further categorized into ‘minima’, ‘intermedia’ and ‘major’ to reflect the severity of disease [3,16]. β-thalassemia major is the most clinically severe disease form and necessitates regular blood transfusions, without which patients are not expected to survive into adulthood. Transfusion-dependent β-thalassemia (TDT) is caused by homozygous or heterozygous mutations in *HBB* which prevent (β0 genotype) or reduce (β+) the synthesis of β-globin [16,17].

Symptoms of severe β-thalassemia typically arise from within a few months of birth and present as pale skin, poor appetite and an increased likelihood of infection in children [18]. The anemia associated with β-thalassemia can cause tiredness, shortness of breath and arrhythmia. If untreated, this may lead to delayed growth, osteoporosis and enlargement of the liver, spleen and heart [16,18]. Patients typically also require chelation to remove excess iron and reduce toxicity [18,19].

### 1.3. Stem Cell Therapy for Haemoglobinopathies

At present, the only cure for SCD and TDT is a stem cell or bone marrow transplant; however, occurrences of this are limited due to the risks associated with rejection and graft-versus-host disease (GvHD) [18,20]. Recently, autologous transplant of genetically modified hematopoietic stem cells (HSCs) and progenitor cells has been shown to promote transfusion independence in TDT patients and this therapy has been approved by the US Food and Drug Administration (FDA) [21,22,23]. The treatment (betibeglogene autotemcel; brand name Zynteglo; Bluebird Bio) involves patient myeloablation and in vitro lentiviral transduction of HSCs with βA-T87Q-globin, followed by autologous transplantation. Overexpression of βA-T87Q-globin compensates for the limited Hb production in β-thalassemia patients and led to transfusion independence in 91% (of 22) of patients, with a median duration of 20.4 months [21]. Although βA-T87Q-globin prevents formation of the polymeric HbS chains which cause RBC sickling in SCD [22], the treatment has not at present been approved by the FDA for the treatment of SCD [23].

#### 1.3.1. Casgevy

In November 2023, the Medicines and Healthcare products Regulatory Agency (MHRA) granted conditional approval to Casgevy (exagamglogene autotemcel; Vertex Pharmaceuticals) for the treatment of sickle cell disease and transfusion dependent β-thalassemia intermedia and major in patients of 12 years and older for whom a human leukocyte antigen-matched related hematopoietic stem cell (HSC) donor is appropriate, but not available. In the case of SCD, Casgevy is licensed for patients with recurrent vaso-occlusive crises who have the βS/βS, βS/β+ or βS/β0 genotype. Casgevy represents a novel therapeutic option for patients suffering with these haemoglobinopathies and is the first treatment worldwide to gain regulatory approval involving genomic editing using the Nobel prize-winning clustered regularly interspaced short palindromic repeats (CRISPR)/Cas9 system in humans. The following sections will discuss the mechanisms of action and regulatory considerations during the approval of Casgevy by the MHRA.

#### 1.3.2. Mechanism of Action

**Figure 1:**
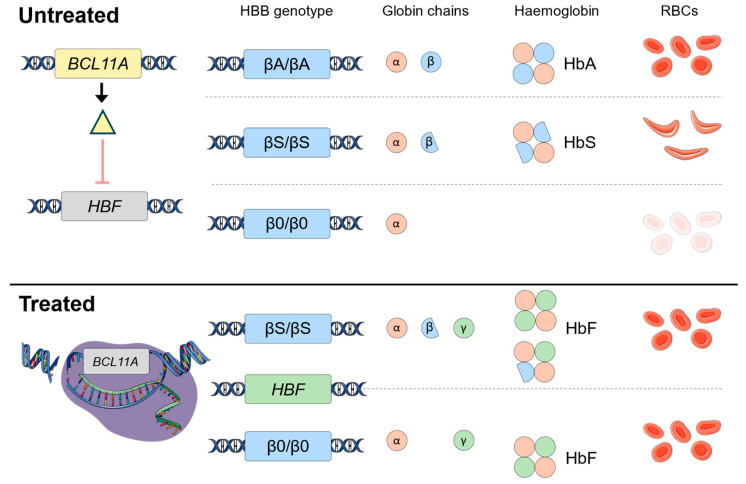
Mechanism underlying development of SCD, TDT and the action of Casgevy. In untreated adults (**top panel**), the gene product of *BCL11A* (yellow triangle) suppresses expression of the *HBF* gene. As such, healthy adults (βA/βA genotype) express normal α- and β-globin chains, leading to production of HbA and healthy erythrocytes. In subjects with SCD (e.g., βS/βS genotype) or TDT (β0/β0 genotype), α-globin expression is normal but β-globin is mutated or extremely limited. This leads to production of pathogenic HbS and RBCs susceptible to sickling, or to a reduced expression of functional Hb. In treatment with Casgevy (**lower panel**), SPY101 (shown in green) in conjunction with Cas9 (purple) causes a double-stranded break in the *BCL11A* promoter region. Although this is subsequently ‘repaired’ by inherent mechanisms, the induced indels serve to downregulate *BCL11A*. As such, γ-globin expression occurs, leading to formation of HbF in TDT and SCD subjects, which compensates for the reduction in HbA found in these patients.

CD34+ HSC and progenitor cells are harvested from the patient and enriched ex vivo. Cells subsequently undergo electroporation in the presence of specific CRISPR single guide ribonucleic acids (sgRNA) and the Cas9 nuclease enzyme. The sgRNA (SPY101-RNP) is complementary to the binding site for GATA1 in the non-coding erythroid lineage-specific enhancer region of the *BCL11A* gene. SPY101-RNP, in complex with Cas9, therefore binds to this enhancer region and executes its endonuclease activity to introduce a double-stranded DNA break. This is repaired by the inherent DNA repair mechanism of non-homologous end joining, which typically produces insertions and deletions in the region. Such mutations to the *BCL11A* enhancer region reduce binding of the transcription factor GATA1, and therefore inhibit expression of *BCL11A*. As *BCL11A* is a potent repressor of γ-globin, this leads to increased γ-globin expression and subsequent formation of HbF. Genetically modified cells are then re-infused into the patient (autologous transplant) to allow in vivo erythropoiesis of RBCs carrying functional HbF (Figure 1).

In patients with SCD, HbF levels constituting ≥20% of total hemoglobin have been shown to be protective against vaso-occlusive crises [24]. Patients with transfusion-dependent β-thalassemia have limited β-globin expression, and therefore low levels of HbA. Filling this absence with γ-globin (and HbF) is therefore predicted to increase levels of circulating Hb and reduce the need for transfusion [21].

## 2. Evaluation of Preclinical Data

From a regulator’s perspective, the role of preclinical development of pharmaceuticals is: (i) to reassure that the claimed benefit of a new product is scientifically justified and (ii) to identify and characterize risks entailed in their use. In case of biotechnology-derived products, their complexity and intricate mechanisms of action often mean that traditional methods for evaluating potential toxicities fall short of comprehensive, and that additional, custom-made approach to examining their safety may be needed.

### 2.1. Proof of Principle

Although naturally occurring only in procaryotes, CRISPR/Cas9 technology can be adapted and introduced into eukaryotic cells to enable a broad range of applications, such as generation of genetically modified cells and animal models [25,26,27,28]. The first such product to enter the clinical world is Casgevy, otherwise known as exagamglogene autotemcel (exa-cel), a cellular product consisting of autologous CD34+ human hematopoietic stem and progenitor cells modified by CRISPR/Cas9-mediated gene editing. As described above, the target gene of Casgevy in these cells is *BCL11A* (B-Cell Lymphoma/Leukemia 11A), a transcriptional repressor of γ-globin in erythroid cells.

Although there are mice that show characteristics of each of β-thalassemia and of sickle cell disease, these mice are immunocompetent and would reject human cells present in Casgevy. Therefore, in the first phase of the preclinical development, pharmacological confirmation of the therapeutic potential of Casgevy did not include either proof of principle studies in such animals, or studies using edited surrogate murine cells. Instead, the sponsor conducted a range of in vitro and in vivo studies to demonstrate the claimed mechanism of action and activity of Casgevy to increase fetal hemoglobin in human autologous CD34+ human hematopoietic stem and progenitor cells. In this respect, the sponsor demonstrated the following:

(A) The guide RNA, SPY101, was able to edit its target *BCL11A* gene, lowering its expression in erythroid cells derived from both healthy donors and patients;
(B) This resulted in increased expression of γ-globin mRNA and protein in edited cells, and thereby increased fetal hemoglobin;
(C) These effects were observable in patient-derived cells;
(D) Edited and differentiated erythroid cells engrafted in vivo in immunodeficient mice persisted up to 20 weeks post-transplant (the longest period evaluated), in blood, bone marrow and spleen. Additionally, the proportion of the various hematopoietic lineages derived from transplanted edited human hematopoietic stem and progenitor cells was not affected by the editing process.

### 2.2. Preclinical Safety

#### Tumorgenicity

Despite the rapid progress in the development of CRISPR/Cas9 technology, fine intricacies of the mechanisms by which the Cas9-gRNA complex recognizes and cleaves its target DNA remain to be fully understood [29]. Despite the targeted design of gRNA, the outcome of CRISPR/Cas9-mediated editing cannot be unquestionably predicted. First, amongst approximately 3 billion base pairs of the human genome, the exclusivity of the binding site for the gRNA cannot be assumed. Amongst others, potential erroneous (off-target) binding and editing of an undesired gene may occur, and in the worst-case scenario, result in activation of otherwise silent oncogenes. Furthermore, the intrinsic cellular mechanisms of DNA repair, which are harnessed to work in concert with CRISPR-editing to re-establish double stranded structure of DNA following Cas9 cleavage, can be erroneous or affected by the CRISPR/Cas9 system itself. Namely, repair of Cas9 cleavage results primarily in small insertions or deletions (collectively called indels). However, due to mechanisms which are still not elucidated, much longer sections of DNA may become deleted [30]. At the far end of this spectrum are rogue genomic rearrangements [31,32] and even catastrophic chromosomal shattering [33].

Given the mode of action of Casgevy, the possibility of tumor formation arising from a genotoxic effect is of particular interest. The first port of call in preclinical development with regards to tumorigenicity of a pharmaceutical are general toxicity studies. In one such study, designed to include evaluations of hematology and clinical chemistry parameters, macroscopic observations and development of tumors, no notable toxicities or tumors were noted in 120 mice injected with Casgevy and followed for 20 weeks. The dose used was ~10-fold higher than the clinical dose and was set as a no observed adverse effect level (NOAEL). HL-60 cells were used as a positive control, which confirmed sensitivity of the method to detect tumors.

Although this study was judged to be a suitable assessment of the safety of the product in vivo, it was not considered sufficient to reach definitive conclusions on genetic toxicity. The standard battery of genetic toxicity tests typically conducted for small molecules is not sufficient to detect the genotoxic potential of CRISPR/Cas9 editing as the editing mistakes could be subtle and take years to exert a pathological effect. Consequently, specialized genotoxicity studies are warranted. For Casgevy, the sponsor designed and conducted a series of additional studies using complementary techniques, to evaluate on-target editing, off-target editing, and the risk for large rearrangements (deletions and translocations).

### 2.3. On-Target Editing

For the precision of on-target editing, the sponsor used long-range polymerase chain reaction (LR-PCR) and hybrid capture next-generation sequencing. This approach can quantify indel formation at the on-target site and infer whether a significant number of translocation events were introduced. Results showed that, in healthy donors, SPY101-RNP editing resulted in an overall rate of on-target indel formation of 88.4%, the majority of indels being shorter than 30 bp in size, which is considered a high precision of editing. In another study, on-target testing was undertaken, using cells from three patients with TDT, three patients with SCD and another four healthy donors. While the size of indels was not determined in this study, the on-target site editing ranged from 60.8% to 71.9% in the patient samples and was similar to that in the samples from healthy donors (49.4% to 61.0%).

### 2.4. Off-Target Editing

In addition to the above-mentioned findings, SPY101-RNP did not cause detectable off-target editing. Translocations after editing with SPY101-RNP were not detected by karyotyping analysis. Potential for off-target activity was further scrutinized by multiple means. The risk of off-target editing was first addressed computationally, using broad nomination of candidate off-target regions. Specifically, in addition to the desired *BCL11A* region, a computational search for so called variant-aware homology regions was performed in order to identify regions of homology, and thus potential binding, between the human genome and the gRNA SPY101. Across the six patients, 249 potential off-target sites were identified using variant-aware homology regions search. Out of these 249 sites, a total of four off-target regions demonstrated a ≥0.2% difference between the untreated and treated indel rate, indicating that those four sites may be similar enough to *BCL11A* to potentially undergo editing. Further analysis showed that all four off-target regions were located in a small intergenic ~500-bp window of the chromosome 3 centromere. Further manual review of these regions showed that they lacked hallmarks of CRISPR/Cas9-induced editing, hence were unlikely to be a result of bona fide editing error.

The results above indicated that therapy with Casgevy caries a low risk of CRISPR/Cas9-mediated genotoxicity. Nonetheless, from a regulatory viewpoint, placing the product on the market introduces additional levels of complexity compared to preclinical and clinical trials. One important consideration is genetic variability, or the presence of polymorphisms, in wider human populations. Indeed, a computational analysis published by Cancellieri and colleagues examined single-nucleotide polymorphism (SNP) in the GATA1 binding motif of *BCL11A* found putative off-target candidates in individuals carrying SNP alleles of *BCL11A* commonly found in African-ancestry populations [34]. The implication of this finding is that allele-specific off-target editing risk may not be equally distributed across populations. Such a consideration warranted a major objection to licensing of Casgevy. In regulatory procedural terms, a major objection is a possible reason to deny approval, if the point is not resolved. The sponsor was asked about the implications of genetic variants enriched in different populations, or even variants present in a given individual, with regards to the risk of off-target editing.

In response, the sponsor provided additional information showing that, in fact, the putative off-target site identified by Cancellieri was included and examined in the sponsor’s preclinical testing. Namely, the computational nomination of off-target candidates conducted by the applicant identified a SNP sequence, rs11451845, that overlapped that identified by Cancellieri et al. Further empirical examination conducted by the sponsor using ultra-deep GUIDE sequencing provided experimental confirmation of the absence of off-target editing by Casgevy at that particular sequence. The above approach was applied to samples from both healthy donors and patients with SCD and TDT, 86% of which were of Black or African-American ancestry.

Overall, despite the suggestion from the publication of Cancellieri et al., testing showed that there was no experimental evidence of an increased risk of off-target editing in either patient population. This was accepted and the major objection was considered as resolved.

### 2.5. Further Considerations

Nonetheless, some other concerns for the regulator remain. For example, once putative off-target sequences are identified, to what extent would it be necessary to determine and report their function in the genome? Clearly, if a putative off-target sequence forms a part of the known protooncogene or a regulator gene, the risk of disrupting it would have to be considered accordingly. In case of Casgevy, the risk sequence rs114518452 was not predicted to have functional consequence, because it is found in an intron of a non-canonical transcript of the gene *CPS1* (an enzyme involved in metabolism of nitrogen which is primarily expressed in the liver) and has no known association with any trait. Nonetheless, these considerations are warranted for all newly identified putative off-target regions. Encouragingly for the future of CRISPR/Cas9-based therapies, a systematic, ultradeep, genome-wide mapping of oncogenic mutation selection during CRISPR/Cas9 editing showed that CRISPR/Cas9 does not introduce or enrich tumorigenic variants in human hematopoietic stem and progenitor cells [35].

Further questions for the regulator include the number of healthy donors and patients which would provide the regulator and the public with sufficient confidence that the risks of gene disruption by Casgevy is appropriately evaluated. Similarly, how many donor samples is required to account for natural variation of engraftment efficacy, given that the long-term efficacy of Casgevy depends partly on engraftment and survival of edited cells in vivo. At present, it is not clear that extending the preclinical examination beyond the above stated number of participants would offer the definitive answer. In such cases, other areas of regulatory process are better placed to provide relevant information by ensuring long-term follow-up of patients that received the therapy. In line with this, the risk management plan upon which Casgevy licensing is dependent contains details on managing delayed platelet engraftment, neutrophil engraftment failure, and gene editing-related oncogenesis [36].

Finally, even a highly precise and desired CRISPR/Cas9 editing is not free of scrutiny and needs to be seen in a wider context of the whole-body function. For example, a targeted gene may be expressed in multiple tissues, or have multiple roles, raising the possibility that its disruption may encompass simultaneously the intended and unintended consequences. In the case in hand, *BCL11A* is expressed in non-hematological tissues, including brain, kidney and gastrointestinal tissues. Favorably for Casgevy, the possibility of *BCL11A* being editing in extra-hematopoietic tissues is effectively precluded by the fact that the entire editing process is conduced ex vivo (i.e., CRISPR/Cas9-SPY101 complex is not systemically injected to patients), hence the exposure of extra-hematopoietic tissues to gene editing machinery is highly unlikely. Nonetheless, it is important to emphasize that CRISPR/Cas9 system is novel, flexible and enables many applications, therefore upcoming CRISPR/Cas9 products should be considered on case-by-case basis, including evaluation of such a possibility.

## 3. Evaluation of Clinical Pharmacology

The role of clinical pharmacology data in regulatory submissions for cell-based therapy is to (i) characterize cell kinetics biodistribution and persistence of the genetically modified cells and the levels of the transgene production; (ii) support the proposed dose and any dose modifications in special populations, such as subjects with impaired renal and hepatic functions; (iii) support any drug interaction recommendations; and (iv) assess pharmacodynamic activity and the relationship between cell kinetics and pharmacodynamics.

### 3.1. Pharmacological Studies

The clinical pharmacology data were derived from the pivotal clinical studies, and therefore no dedicated clinical pharmacology studies were conducted. The lack of clinical pharmacology studies was not raised as a concern as the European Medicines Agency requests dedicated clinical pharmacology studies only when feasible with the aim of monitoring cell viability, proliferation, body distribution and function, and this guidance is followed by the MHRA. The guidance also indicates that alternative approaches in the clinical development plan could be acceptable. For Casgevy, the persistence and pharmacodynamic data were recorded in the pivotal clinical studies, which was considered sufficient to confirm the clinical pharmacology aspects of Casgevy treatment.

The following pharmacodynamic endpoints were evaluated in three studies, which aimed to establish efficacy and safety in subjects with TDT (study 111) and SCD (study 121) during a period of two years and during longer-term follow-up (study 131):

(A) Persistence of the edited cells (proportion of alleles with intended genetic modification by in peripheral blood and in CD34+ cells of the bone marrow);
(B) Absolute and fractional values of HbF.

### 3.2. Persistence of the Edited Cells

Persistence of edited cells was measured as the mean proportion of alleles with the intended genetic modification in the CD34+ cells of the bone marrow and in peripheral blood. It was 77.85% and 86.04% in subjects with TDT and SCD, respectively, at month 6 in bone marrow. In peripheral blood, it was maintained at higher than or equal to 60% and 70% in subjects with TDT and SCD, respectively, from the second month onward, throughout the duration of follow-up in both study 111 and in study 121. The bone marrow data were derived from a limited number of subjects, limited time points, and with no baseline measurements, making it difficult to draw a conclusion about the expansion and persistence of the CD34+ cells with intended genetic modification.

**Figure 2:**
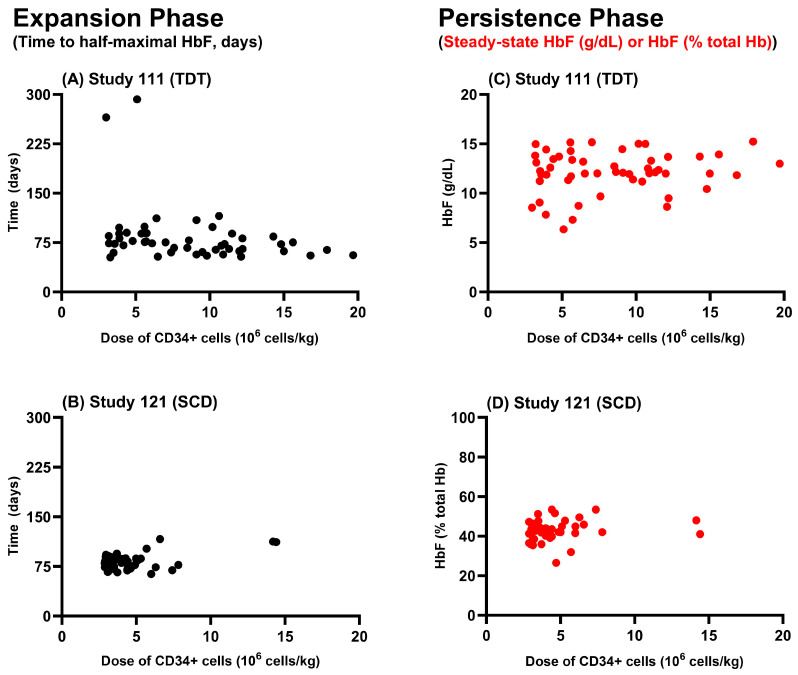
Dose–response relationship for expansion and persistence of HbF in Study 111 and Study 121. Dose (CD34+ cells/kg) versus expansion time (**A**,**B**) and versus persistence quantified as steady state HbF in g/dL (**C**) or as a percentage of total HbF (**D**) for TDT (**A**,**C**) and SCD (**B**,**D**) patients. Data from UK PAR [36].

The data derived from peripheral blood showed trends for expansion (time to half maximum HbF) and persistent (steady state HbF) phases. The sponsor was asked to submit further analysis to show the relationship between starting dose and these two phases (Figure 2). Figure 2 shows that there was no observed relationship between Casgevy dose (weight normalized CD34+ cells measured in 10^6^ cells/kg) and either the expansion or the persistence phases. This indicates that the range of the starting doses used in the clinical studies 111 and 121 provided comparable expansion and persistence of the CD34+ cells in the plasma of all subjects. Although for many medications it is recommended to use the lowest efficacious dose, in the case of autologous transplantation, higher doses are not associated with safety concerns (see clinical data below) and have been shown to reduce variability in cell kinetics. Improved outcomes have previously been associated with doses of >5 × 10^6^ CD34+ cells/kg following similar procedures during cancer treatments [37]. Taking studies 111 and 121 together, 14% of subjects administered ≤5.5 × 10^6^ CD34+ cells/kg did not meet the primary outcomes, whilst 0% of those administered >5.5 × 10^6^ CD34+ cells/kg failed to meet these, supporting the use of a higher dose where possible.

### 3.3. Foetal Haemoglobin

**Figure 3:**
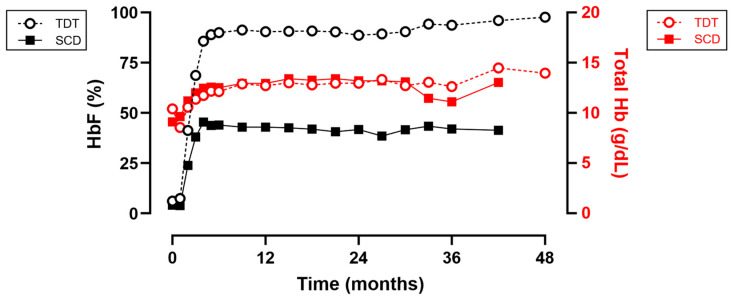
Effect of Casgevy on hemoglobin. Volume of total Hb (g/dL; red) and proportion of Hb that is HbF (%; black) for TDT (circles) and SCD (squares) subjects. Data are shown for studies 111, 121 and 131. Data from UK PAR [36].

In all studies, total Hb, total HbF and HbF % were increased significantly by month 3 and plateaued from month ~6 onward throughout the duration of follow-up, with this persisting into in study 131 (Figure 3). At month 3, mean (SD) total Hb levels were increased from 10.40 and 9.11 g/dL to 11.40 (2.26) g/dL and 11.99 (1.42) g/dL in study 111 and 121, respectively, and were maintained with mean ≥12 g/dL from month 6 through the duration of follow-up. Mean HbF levels were increased by month 3 from a negligible proportion and maintained at ≥10 g/dL and ≥4.0 g/dL for study 111 and 121, respectively, from month 6 through the duration of follow-up (Figure 3). Mean proportion of total Hb comprised by HbF (HbF %) was maintained with mean ≥86% and ≥40% for study 111 and 121, respectively, from month 6 through the duration of follow-up (Figure 3).

These data agree well with the preclinical proof-of-principle described above and confirm the mechanism of action of treatment with Casgevy. The lower levels of HbF% in SCD as compared to TDT subjects was justified as HbF in SCD subjects would compete with endogenous βS-globin production and that circulating RBCs would express approximately 30% to 50% HbF, whereas TDT subjects lack meaningful β-globin production from the endogenous mutated β-globin gene, and therefore near 100% HbF could be achieved.

The recommended dose of Casgevy is at least 3.0 × 10^6^ CD34+ cells/kg IV for subjects with TDT or SCD aged 12 years and older. This weight-based treatment is consistent with accepted safe clinical practices regarding the dose of CD34+ cells required to achieve durable long-term hematopoietic reconstitution following autologous transplantation [38,39,40]. The dose rationale was well supported as the proposed dose did not lead to toxicity or a lack of efficacy. Rather, this regimen typically resulted in successful neutrophil and platelet engraftment, which caused an increase in both total Hb and in HbF from baseline, leading to improved clinical outcomes.

### 3.4. Dosage for Special Populations

To address the special populations (e.g., subjects with renal or hepatic impairment, pediatric or old age, high or low body weight) in TDT and SCD subjects, the sponsor presented subgroup analyses for selected endpoints such as allelic editing and HbF levels. The populations tested were age groups at screening (≥12 and <18 years of age; and ≥18 and ≤35 years of age), genotypes for study 111 (β0/β0-like and non-β0/β0-like), genotypes for study 121 (βs/βs, βS/β0 and βS/β+), sex groups (male and female), median body weight at baseline for study 111 (≤52 kg and >52 kg) and median body weight at baseline for study 121 (≤67 kg and >67 kg). The overlapping 95% CI across all timepoints for all compared subgroups indicated that there was no impact of age, genotype, sex or body weight on the selected endpoints and no dose adjustment was needed based on these intrinsic factors. However, sample number in the subgroups was small, and therefore no firm conclusion can be drawn.

Casgevy is indicated in subjects aged ≥12 yeas. In the full analysis dataset for TDT and SCD, outcomes for both adolescents (≥12 to <18 years of age) and adults (≥18 to ≤35 years of age) were similar with the overall results from the main analyses for each endpoint, indicating that the Casgevy mechanism of action is independent of age.

### 3.5. Myeloablative Treatment

Busulfan was used as single agent for myeloablative treatment which aims at lowering the number of stem cells in the bone marrow to allow the genetically modified cells to flourish. Myeloablative bone marrow conditioning in Casgevy studies utilized 3.2 mg/kg busulfan once daily (qd) for four consecutive days or 0.8 mg/kg every 6 h (q6h), with dose adjustment being made based on PK from days 1 to 3. All subjects achieved profound neutropenia, demonstrating the acceptability of the protocol. Caution should be taken with dose adjustments as busulfan has a narrow therapeutic window. These regimens have been established previously for other treatments which depend upon myeloablation.

**Figure 4:**
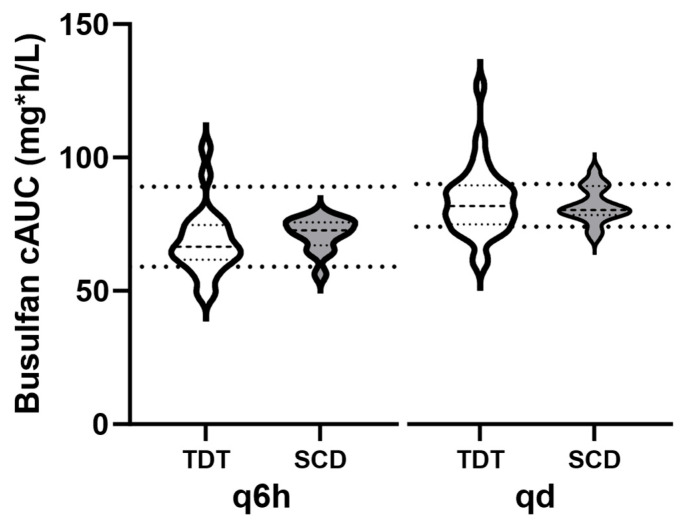
Busulfan cAUC for each dosing regimen. Median and quartile values are shown within each violin plot for TDT and SCD cohorts. The outer dashed lines represent the target range for each regimen. Data from UK PAR [36].

As shown in Figure 4, the protocol-specified busulfan cAUC was within the target range in 74% (*n* = 23) and 52% (*n* = 25) of TDT subjects on the q6h and qd regimens, respectively, and was achieved in 96% (*n* = 23) and 58% (*n* = 12) of SCD subjects on the q6h and qd regimens, respectively. These data confirmed that the two busulfan regimens, either as qd or q6h, provided exposure which was within the target AUC for most subjects. Although the q6h regimen appeared more successful at maintaining busulfan AUC within the target range, all subjects had successful engraftment, which provided further evidence for the adequacy of both busulfan regimens.

## 4. Statistical Evaluation

Assuring reliability of study results is key aspect of statistical evaluation. Therefore, there is a requirement for single-arm studies to pre-define a detailed statistical analysis plan before commencement. The study design, data collection and analysis methods can all affect the ability to estimate the desired treatment effect reliably or its interpretation. Understanding the exact research question being answered in a study is essential for an appropriate interpretation of results. The estimand framework (ICH E9 (R1) [41]) includes key information essential to the proper interpretation of the study results (e.g., study withdrawal, treatment discontinuation, death).

For Casgevy, the pivotal studies had similar design (both studies were single-arm, open-label studies) and used group sequential design to facilitate the conduct of interim analyses. According to ICH E17 guideline, the sample size should be sufficient to enable the evaluation of the overall treatment effect, assuming that the effect is applicable to the whole target population [42]. Both studies were adequately powered (power was more than 90%) to address the primary objective of the study. In both studies, sample size was determined by investigating the operating characteristics of the efficacy boundaries under different scenarios of the true response rate for the primary and key secondary efficacy endpoints.

The familywise type I error rate was controlled by an alpha spending approach for tests at interim and final analyses, and a sequential testing of the primary and key secondary efficacy endpoints. The alpha spending approach allows the timing of the interim analyses to be flexible. The stopping criterion for superiority were calculated as a function of the amount of information present at the time of the interim analysis. Control of the probability of false positive conclusions at the study level is an important consideration for regulatory decision making.

Both studies were designed to evaluate efficacy based on the Primary Efficacy Set (PES) and the Full Analysis Set (FAS). The FAS included a subset of the Enrolled Set that includes subjects who have received Casgevy infusion. PES included a subset of FAS that includes all subjects who had been followed for at least 16 months post Casgevy infusion and for at least 14 months after completion of the RBC transfusions for post-transplant support or TDT management (Study 111) or SCD management (Study 121).

Ideally, efficacy should be described from time of enrolment since subjects could die or withdraw from the study or the manufacturing of the cell may not be successful while waiting for Casgevy infusion. Therefore, all subjects that entered the studies upon providing informed consent should be used as the primary analysis set.

Two subjects withdrew from study 111 (TDT) for reasons unrelated to treatment. In study 121 (SCD), 14/63 (22%) enrolled subjects discontinued prior to infusion (five before mobilization and nine after mobilization but before conditioning, of which five/nine were discontinued due to inadequate cell collection). No subject discontinued due to AE.

For the primary analysis, both studies used exact Clopper–Pearson method to derive 95% confidence interval of the estimated proportion of subjects achieving. Statistical testing was used to determine whether the treatment effect is above a prespecified threshold, which is reflected in whether the null hypothesis related to the threshold is rejected. Hypothesis testing was based on 2.5% one-sided *p*-value (against a null hypothesis of 50% response rate).

There were no treatment discontinuations as Casgevy was administered as a single-dose IV infusion on Day 1. Subjects who died or discontinued the study were handled differently depending upon whether the reasons for study discontinuation were related to adverse events due to Casgevy and upon length of follow-up. The potential for bias in open label studies was considered by the assessors, and this is discussed in more detail below (also see [43]). A number of measures were implemented in the submitted studies to minimize risk of bias including the use of central laboratories for hemoglobin assessments for the baseline characteristics and efficacy analyses and the use of an independent endpoint adjudication committee to adjudicate transfusion events (TDT) and VOC events (SCD), both historical as well as after Casgevy infusion. Missing data were not imputed. For subjects who were lost to follow-up or death, safety and efficacy analyses were based on their available data before death or loss to follow-up.

## 5. Evaluation of Clinical Data

Statistical inference of clinical trials is needed to verify the efficacy and safety of medicinal products. A randomized, blinded, controlled trial is widely accepted as the best design to evaluate the efficacy of a new treatment because randomization reduces bias, balances covariates and permits a valid test of significance. Large numbers of subjects are included in such trials in order to generate robust data on efficacy. There are, however, situations where randomized controlled trials may not be feasible or practical owing to ethical considerations [44] and evidence from single-arm trials may be all that can be obtained. In such cases, regulators must use alternative methods in order to assess the efficacy of treatments [43]. In the claim for the indication in TDT and for SCD, one single-arm trial was submitted per disease, i.e., the trials were not randomized, blinded or controlled.

### 5.1. Clinical Data

The company submitted a single-arm demonstration study that enrolled subjects with transfusion-dependent β-thalassemia (study 111) or SCD (study 121). The baseline characteristics of subjects in these cohorts are shown in Table 1, along with the Casgevy dose received and follow-up duration for each cohort.

For the primary efficacy TDT cohort, 39/42 subjects achieved the primary outcome by maintaining an average Hb ≥9 G/dL without red blood cell transfusions for at least 12 consecutive months any time after Casgevy infusion. For those who achieved the primary outcome, the median time to achieve ‘free from transfusion’ was ~1 month, the maximum time was ~3 months and the total duration of being transfusion-free ranged from 13 to 24 months. Mean HbF increased from trace concentrations at baseline to 10.8 G/dL by month 6 and remained so for the duration of follow-up. Adverse events were, in the main, those known to be associated with autologous stem cell transplants and so may be anticipated and managed appropriately.

For the primary efficacy SCD cohort, 28/29 subjects achieved the primary outcome by not experiencing any severe vaso-occlusive crises for at least 12 consecutive months, with 27 of these remaining crisis-free until the end of study date or the interim data cut date (whichever was earlier). The other participant was crises-free for ~23 months after Casgevy infusion and subsequently did not experience another crisis during the study period. For the 28 subjects who did achieve the primary endpoint, the mean (SD) vaso-occlusive crisis free duration was 18.3 (3.4) months. The subject who continued to experience crises suffered eight events between 12.1 and 21.2 months after infusion, indicating there was no reduction in crises for this subject. Adverse events were, in the main, those known to be associated with autologous stem cell transplants and so may be anticipated and managed appropriately.

A single-arm demonstration study has been conducted in the context of a rare disease, where the disease is stable/progressive and with the caveat of the fallacy of human reasoning referred to as post hoc ergo propter hoc (Latin: ‘after this, therefore because of this’). An interim report has been submitted. Outcomes of the study are likely biased because: (i) the trial had a single-arm design (i.e., without an internal control) and (ii) the trial relied on comparison with the need for red blood cell transfusion in each subject in the preceding year before exposure to Casgevy.

Long-term maintenance of efficacy beyond 36 months has not yet been established. Three TDT subjects have not achieved freedom from red blood cell transfusion (yet have achieved >80% reduction in the need for red blood cell transfusion). None of the SCD subjects, including the one who failed to meet the primary endpoint, required RBC transfusions for sickle cell disease-related indications starting 12 months after infusion. All SCD subjects receiving Casgevy (including the one who did not meet the primary endpoint) remained free from hospitalization for at least 2 months after transfusion. Overall, the claims that Casgevy is indicated to treat people with TDT or SCD is considered supported by the results of the submitted study.

### 5.2. Assessment of Clinical Data

A single-arm study was submitted in order to support the claims of the company. Inferential statistics may be used to compare the differences between treatment groups, yet, within the setting of a single-arm non-randomized study, they are unable to provide valid probability statements about treatment effects owing to their inability to control for bias; single-arm studies are unable to support the assumptions that are associated with randomization. Additional details on the interpretation of these single-arm studies have recently been published, making use of the scheme described by Toulmin coupled to an analysis of causal inference. [43]. Conventional statistics may be used in a descriptive manner with a detailed description of the parent population, yet this would lose the connection to a causal difference in outcome.

People with TDT and SCD require frequent transfusions of red blood cells and will develop symptoms and consequences of iron overload; with SCD subjects additionally experiencing crisis events. For these reasons, the main claim that Casgevy can be used to treat people with TDT or SCD would be desirable if those treated no longer needed transfusions of red blood cells. In the context of a single-arm study, a binary outcome (subjects either achieved or did not achieve freedom from the need for red blood cell transfusions/freedom from vaso-occlusive crises) is preferred to a continuous outcome (that may be affected by individual variation) or a time-to-event outcome (where there is not a comparator). Time-to-event endpoints are unsuitable because it is generally difficult to attribute these events to the treatment. There are also difficulties with determining the starting point of being at risk (i.e., time 0). Causal attribution of treatment effect is challenging for continuous outcomes as they are subject to change and random fluctuation over time due to within-subject variability.

At this stage in product development, where 39/42 TDT subjects have become free of the need for transfusions of red blood cells within weeks of administration of Casgevy and 28/29 SCD subjects were free of vaso-occlusive crisis [36], the evidence is found to be dramatic, credible and accurate. The outcome is unlikely to have occurred spontaneously or to have been influenced by other clinical management or alternative therapies.

Aspects of clinical safety have been found to be related, in the main, to the autologous transplantation procedure; it is acknowledged that unknown elements of safety may yet be revealed by long-term follow-up of recipients of Casgevy. Durability of efficacy is, at present, also an unknown that may also be ascertained by long-term follow-up of recipients. Overall, the benefit risk of Casgevy is found to be positive.

## 6. Conclusions

A total 83 subjects were dosed with Casgevy, including 48 subjects with TDT and 35 subjects with SCD. The data presented by the sponsor was judged to be reliable and supportive of their claims for the efficacy of Casgevy. The novel mechanism of action was well supported by the preclinical data, and concerns surrounding off-target editing were adequately addressed by in vitro and in silico work. Clinically, the Casgevy dosages used by the sponsor were shown to dramatically improve clinical outcomes over the study duration.

The sponsor sought a conditional national license for Casgevy. Conditional marketing authorization may be granted by the MHRA for medicinal products which fulfil an unmet medical need. In these instances, it is accepted that comprehensive clinical data may not be complete at the time of approval, but it necessitates that sponsors will continue to collect such data post-authorization for annual review as part of the post-approval obligations (i.e., conditions) to confirm the long-term efficacy and safety of Casgevy. Casgevy was granted orphan drug designation for treatment of both SCD and TDT due to the low prevalence of both diseases, supporting the notion that large clinical studies may be challenging for the sponsor. It was therefore considered that although the number of subjects exposed to Casgevy is small, further subgroup analyses by region did not reveal any clinically relevant difference attributable to Casgevy based on geographical region, which supports the generalizability of the study’s findings. In addition, further information on the clinical safety and efficacy can be sufficiently addressed using a robust risk management plan (RMP) [36]. The RMP aims to collect further data which can be used to evaluate risks that may not have been fully satisfied by the provided data, such as the risk of gene editing-related oncogenesis and the long-term safety and efficacy of Casgevy. Such data collection will also allow for further comparisons to be drawn between the efficacy of Casgevy and existing standard treatment regimens. According to Casgevy RMP, enrolled patients in Casgevy clinical studies will be followed-up for up to 15 years.

Following careful evaluation of the evidence, the benefits of Casgevy for the treatment of SCD or TDT were deemed to outweigh the risks. Casgevy has therefore been licensed for the treatment of SCD or TDT in patients 12 years of age and older for whom hematopoietic stem cell transplantation is appropriate and a human leukocyte antigen-matched related hematopoietic stem cell donor is not available. In the SCD indication, patients experiencing recurrent vaso-occlusive crises and who have the βS/βS, βS/β+ or βS/β0 genotype may be eligible for treatment.

The conditional licensing of Casgevy by the MHRA represents a first-in-class approval for the treatment of two diseases which bring significant personal and economic healthcare burdens. Following approval by the MHRA, other regulatory bodies have followed suit and approved Casgevy for use. The major similarities and differences in the decision making between regulatory agencies have been previously discussed [45]. It is hoped that this work will pave the way for development of future novel treatments, both in the UK and globally.

## Figures and Tables

**Table 1:** Characteristics of subjects involved in study 111 (TDT) or study 121 (SCD).

	TDT	SCD
No. of adult participants (≤35 years)	29	23
No of. pediatric participants (≥12 to <18 years)	13	6
Median age (years)	20	21
Proportion of females (%)	50	44.8
TDT-related RBCs transfused (median [range], units/year)	35 [20.5–71.0]	3.5 [0–75.5]
Genotype	β0/β0-like: 25/42	βS/βS: 28/29 βS/β0: 1/29
Dose (median [range], ×10^6^ CD34+ cells/kg)	7.5 [3.0–19.7]	4.0 (2.9–14.4)
Follow-up duration (median [range], months)	23.9 [16.1–27.1]	23.6 [16.1–25.6]

## Data Availability

No new data were created or analyzed in this study. Data sharing is not applicable to this article.
